# A Comparison Study of Sampling and Analyzing Volatile Organic Compounds in Air in Kuwait by Using Tedlar Bags/Canisters and GC-MS with a Cryogenic Trap

**DOI:** 10.1100/tsw.2006.111

**Published:** 2006-05-12

**Authors:** Hongmao Tang, Khaliq R. Beg, Yousef Al-Otaiba

**Affiliations:** Coastal and Air Pollution Department, Urban and Environment Division, Kuwait Institute for Scientific Research, P.O. Box 24885, 13109 Safat, Kuwait

**Keywords:** volatile organic compounds, analysis, Tedlar bag, SUMMA canister

## Abstract

Kuwait experiences desert climatic weather. Due to the extreme hot and dry conditions in this country, some analytical phenomena have been discovered. Therefore, a systematic study of sampling and analyzing volatile organic compounds in air by using GC-MS with a cryogenic trap is reported in this paper. This study included comparisons of using different sample containers such as Tedlar bags and SUMMA canisters, and different cryogenic freezing-out air volumes in the trap. Calibration curves for different compounds and improvement of replicated analysis results were also reported here. The study found that using different sample containers produced different results. Analysis of ambient air samples collected in Tedlar bags obtained several volatile organic compounds with large concentrations compared to using SUMMA canisters. Therefore, to choose a sample container properly is a key element for successfully completing a project. Because GC-MS with a cryogenic trap often generates replicated results with poor agreement, an internal standard added to gas standards and air samples by using a gas syringe was tested. The study results proved that it helped to improve the replicated results.

## INTRODUCTION

Ambient and indoor airs contain a large number of volatile organic compounds (VOCs) at levels that exhibit ecological and health effects. The role and importance in atmospheric chemistry of organic compounds produced by human activity was recognized by Haagen-Smith about 50 years ago[1]. The VOCs with 1–11 carbons in the molecules are precursors required for the photochemical production of ozone in the atmosphere. The very important thing to human beings is that many VOCs are individually carcinogenic and mutagenic in their own right. VOCs are also greenhouse gases. Under the UN Framework Convention on Climate Change on December 4, 1997, they should be controlled. Therefore, monitoring VOC concentrations in air has become a very important issue in environmental protection.

Health effects of VOCs have focused on benzene, which is a carcinogenic compound even if at very low concentrations (sub-ppb level). It is thought that benzene and 1,3-butadiene exposure may account for around 10% of leukemia incidence across the U.K.[2]. It has been recognized that the benzene added to unleaded petrol to maintain vehicle performance may be a worse threat to human health than the lead that it displaced[2].

Concern about public health and the correct assessment of the factors associated with visibility impairment by organic compounds have produced a concerted effort throughout the world to develop sampling and analytical methods in air.

The demand for accurate, reliable, and sensitive techniques for monitoring trace levels of VOCs has been an issue for further research and improvement due to their volatility and small concentration in ambient air for a long time. The selection of an optimal sampling method for target compounds depends greatly on the physicochemical nature of these compounds and their expected concentrations in the air.

The most important physical properties governing the design of a sampling method for VOCs from air are vapor pressure and polarity. VOCs are compounds whose vapor pressures are ranged less than $10^{-5}$ KPa[3]. Over the past few decades, many methods for monitoring VOCs in air have been developed and subsequently improved. Those methods include active integrative method, passive integrative method, and active real-time analytical method or semi-real-time analytical method. Semi-real-time methods employ various types of bags, canisters, and adsorbent cartridges to collect samples that may then be analyzed in the field by a variety of techniques such as gas chromatograph (GC) or GC with mass spectrometry (MS). The majority of methods that have been developed for ambient VOC monitoring have been either container based (such as a canister) or sorbent based[4]. Canister sampling involves the collection of the whole air matrix in a precleaned evacuated collection device. This method is very useful for the most volatile species like C2 – C3 hydrocarbons. The sorbent method normally uses a pump to sample air samples. The common adsorbents include activated carbon, porous polymers, molecular sieve, silica gel, etc.

The canister includes SUMMA canisters and specially prepared canisters[5]. They can be used for collecting air samples over the atmospheric pressure (pressurized) or below the atmospheric pressure (subatmospheric pressure). Many of the VOCs in the USEPA Method TO-15 have been tested for stability in concentration when stored in canisters under conditions typical of those encountered in routine ambient air. Actually, the stability of these compounds under all possible conditions is not known[6]. A model to predict compound losses due to physical adsorption of VOCs on canister walls and to dissolution of VOCs in water condensed in the canisters was developed by Coutant[7]. The losses of VOCs in the canister can be generally classified as physical adsorption, chemical reaction, aqueous hydrolysis, and biological degradation. Unfortunately, there is no model that can be used to estimate all these losses.

Canister-based samplers have been evaluated by Jayanty[8]. The study found that the canister was an efficient means for the collection of some selected volatile and toxic compounds. The selected organic compounds could be stable over a period of 2 weeks. Numerous compounds, many of which are chlorinated VOCs, have been successfully tested for storage stability in pressurized canisters[9,10]. However, minimal documentation is currently available demonstrating stability of VOCs in subatmospheric pressure canisters.

Tedlar bags have been routinely used for collection of air samplers for many years and the Tedlar bag sampling system is one of the recommended sampling techniques cited in USEPA Reference Method 18[11]. The applicability of Tedlar bag sampling systems for collection and analysis of VOCs was studied by McGaughey et al.[12]. Pau et al. introduced a new, smaller (5–7 l) Tedlar bag in 1991[13], and showed that smaller Tedlar bags can be used effectively for sampling many toxic organic compounds and that the small bags have similar relative standard deviations in comparison to the large ones. The big drawback for the Tedlar bags is the loss of smaller molecular VOCs such as $C_2$ during storage.

Gas chromatography equipped with a mass spectrometer as detector (GC-MS), because of its high sensitivity and selectivity for VOCs, has been incorporated into cryogenic traps for VOC analysis. A cryogenic trap normally is packed with small particles such as glass beads in order to increase contact surfaces. When gaseous samples pass the trap, the sample flow cannot keep the same pathway from run to run. If the sample flow is closer to the trap wall, the trapping efficiency will be higher than in the trap center since the trap is surrounded by liquid nitrogen and the walls directly contact the liquid nitrogen. Thus, trapping efficiencies from run to run will be different. Therefore, due to the operation nature in the system, the replicate analytical results always have some large deviations. Average results for the replicated analysis are normally used in the EPA TO14 and TO15 methods.

Kuwait lies at the northwest corner of the Arabian Gulf, between latitudes of 28 and 30 N and between longitudes 46 and 48 E. Most of the Kuwait mainland is a flat sandy desert, gradually sloping down from the extreme west of Shigaya and Salmi (300 m high) towards sea level in the east. Therefore, the weather in summer in Kuwait is extremely hot and has fewer rains. The photochemical reactions in this area are expected to be really active. We used SUMMA canisters and Tedlar bags to sample VOCs in the same outdoor location, and found different compounds from air samples in canisters and Tedlar bags. In order to find the reasons, a systematic study of VOC analysis including sampling methods, cryogenic trapping volume, replicate analysis with and without internal standard, etc. was conducted. The findings are reported in this paper.

## EXPERIMENTAL PROCEDURE

### Sampling Method

Three different sampling methods were employed in this study, which include SUMMA canister, Tedlar bag, and activated carbon tube.

- **SUMMA Canister** — 6-l SUMMA stainless steel canisters (Entech, USA) were used. Before sampling, the canister was cleaned and evacuated by a canister clean system (Entech, USA). Subatmospheric pressure procedure was employed in this study.
- **Tedlar Bag** — 1- and 5-l Tedlar bags were used. The Tedlar bags were cleaned as follows. Each Tedlar bag was filled with zero air and heated above an oven at about 50$^{o}$C for 5 min. Then, the Tedlar bag was evacuated. This procedure was repeated 10 times.
- **Activated Carbon Tube** — The activated carbon tubes were supplied by SKC, Canada. The carbon was packed in two sections in the tube separated by glass wool in the middle.

### VOC and Internal Standards

In this study, 39 different VOC mixtures (Scott-Marrin, Inc. USA) kept in a cylinder were used as standards. The compound names, formulas, and molecular weights are listed in Table 1. With pure air (zero air), each VOC was diluted to three concentration levels in Tedlar bags: 5, 25, and 50 ppb.

2-Methoxy-2-methyl propane (Fisher, USA) was used as internal standard. The compound was first diluted to 42 ppm in a Tedlar bag as a stock standard. Then 1 ml of the stock standard was injected into a Tedlar bag filled with air samples or calibration standards by using a gas syringe.

### Analytical Device

A GC-MS system (Hewlett Packard, USA, Model G1800A) equipped with a preconcentrator (Entech,USA, Model 7000) was used for analysis of air samples in canisters and Tedlar bags. The GC-MS was operated in scan mode. A fused silica capillary column (60 m $\times$ 0.2 mm I.D., 1.1-µm film thicknesses) was used in the GC. A temperature program was set up as follows: initial temperature 35$^{o}$C; initial time 3 min; temperature from 35 to 95$^{o}$C at 15$^{o}$C/min, then to 105$^{o}$C at 2.5$^{o}$C/min; finally to 205$^{o}$C at 5$^{o}$C/min, and at 205$^{o}$C for 5 min.

**TABLE 1**
**List of VOC Concentrations (Volume Fraction) in the Standard Mixture**

| SR No. | Name of Compounds | Concentration | Mol. Wt. | Formula |
|---|---|---|---|---|
| 1 | Chlorodifluoromethane | 50 ppb | 86.5 | $CHClF_2$ |
| 2 | n-Butane | 50 ppb | 58.08 | $C_4H_{10}$ |
| 3 | Butane, 2-methyl | 50 ppb | 72.15 | $C_5H_{12}$ |
| 4 | Furan | 50 ppb | 68.07 | $C_4H_4O$ |
| 5 | n-Pentane | 50 ppb | 72.14 | $C_5H_{12}$ |
| 6 | Dichloromethane | 50 ppb | 84.93 | $CH_2Cl_2$ |
| 7 | Ethane, 1,1,2-trichlotrifluoro | 50 ppb | 187.38 | $C_2Cl_3F_3$ |
| 8 | Cyclopentane | 50 ppb | 70.134 | $C_5H_{10}$ |
| 9 | Acetone | 50 ppb | 58.07 | $C_3H_6O$ |
| 10 | Pentane, 2-methy | 50 ppb | 86.17 | $C_6H_{14}$ |
| 11 | 2-Methyl-1-pentene | 50 ppb | 84.16 | $C_6H_{12}$ |
| 12 | Trichloromethane | 50 ppb | 119.38 | $CHCl_3$ |
| 13 | n-Hexane | 50 ppb | 86.176 | $C_6H_{14}$ |
| 14 | Methylcyclopentane | 50 ppb | 84.16 | $C_6H_{12}$ |
| 15 | Tetrachloromethane | 50 ppb | 153.82 | $CCl_4$ |
| 16 | Ethanol | 50 ppb | 46.06 | $C_2H_6O$ |
| 17 | 2-Butanone | 50 ppb | 72.106 | $C_4H_8O$ |
| 18 | Benzene | 50 ppb | 78.11 | $C_6H_6$ |
| 19 | Cyclohexane | 50 ppb | 84.16 | $C_6H_{12}$ |
| 20 | 3-Methylhexane | 50 ppb | 100.203 | $C_7H_{16}$ |
| 21 | n-Heptane | 50 ppb | 100.2 | $C_7H_{16}$ |
| 22 | Methylcyclohexane | 50 ppb | 98.18 | $C_7H_{14}$ |
| 23 | 1,4-Dioxane | 50 ppb | 88.106 | $C_4H_8O_2$ |
| 24 | 2-Pentanone | 50 ppb | 86.13 | $C_5H_{10}O$ |
| 25 | 2,4-Dimethylhexane | 50 ppb | 114.23 | $C_8H_{18}$ |
| 26 | Toluene | 50 ppb | 92.14 | $C_7H_8$ |
| 27 | 3-Methylheptane | 50 ppb | 114.23 | $C_8H_{18}$ |
| 28 | Ethylbenzene | 50 ppb | 106.167 | $C_8H_{10}$ |
| 29 | p-Xylene | 50 ppb | 106.17 | $C_8H_{10}$ |
| 30 | m-Xylene | 50 ppb | 106.17 | $C_8H_{10}$ |
| 31 | o-Xylene | 50 ppb | 106.17 | $C_8H_{10}$ |

**TABLE 1 (CONTINUED)**

| | | | | |
|---|---|---|---|---|
| 32 | 1-Ethyl-3-methylbenzene | 50 ppb | 120.2 | $C_9H_{12}$ |
| 33 | 1-Ethyl-4-methylbenzene | 50 ppb | 120.2 | $C_9H_{12}$ |
| 34 | 1,3,5-Trimethylbenzene | 50 ppb | 120.2 | $C_9H_{12}$ |
| 35 | 1-Ethyl-2-methylbenzene | 50 ppb | 120.2 | $C_9H_{12}$ |
| 36 | 1,2,4-Trimethylbenzene | 50 ppb | 120.2 | $C_9H_{12}$ |
| 37 | n-Decane | 50 ppb | 142.28 | $C_{10}H2_2$ |
| 38 | 1,2,3-Trimethylbenzene | 50 ppb | 120.2 | $C_9H_{12}$ |
| 39 | n-Dodecane | 50 ppb | 170.337 | $C_{12}H_{26}$ |

The VOCs collected in the canisters and Tedlar bags were enriched by passing a known volume of air through a trap in the preconcentrator using cryogenic preconcentration and cryofocusing. The sample was finally transferred to the GC.

A GC-MS system (Agilent Technologies, USA, Model 5973) was used for analysis of VOCs collected in the activated carbon tube. After collection, the carbon in the tube was extracted with dichloromethane. The extract was injected into the GC-MS system by using a syringe.

Both GC-MS systems had a Wiley 138 library for identifying VOCs.

## RESULTS AND DISCUSSION

### VOCs in Canisters and Tedlar Bags

Basically, the ambient air VOCs found in the canisters and Tedlar bags were almost the same at the same location in Kuwait. But, it was a surprising finding that several high-concentration VOCs (such as VOCs at retention time 18 and 23 min, hereby named as UVOC) found in the Tedlar bags had very small peaks in the canister samples (see Figs. 1 and 2). In order to further confirm the findings, air samples were also collected outdoors and indoors by Tedlar bags, and outdoors by an activated carbon tube. The analytical results from indoor and outdoor air samples in the Tedlar bags clearly showed that UVOC did indeed exist in the ambient air (see Fig. 3).

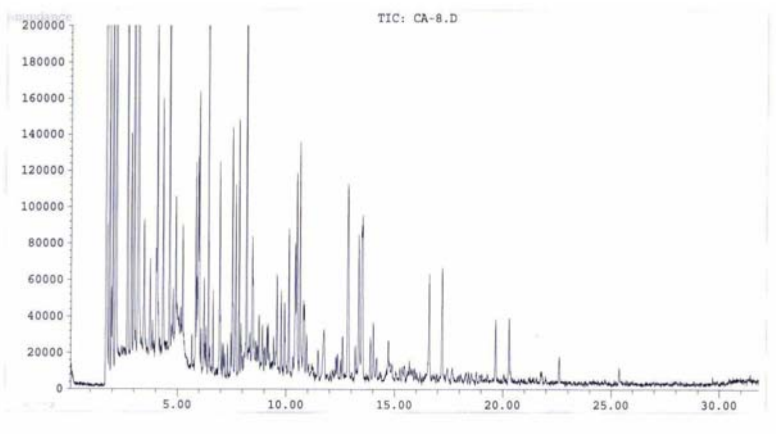

**FIGURE 1**. Total ion chromatogram of an air sample collected in a canister, preconcentration volume 300 ml.

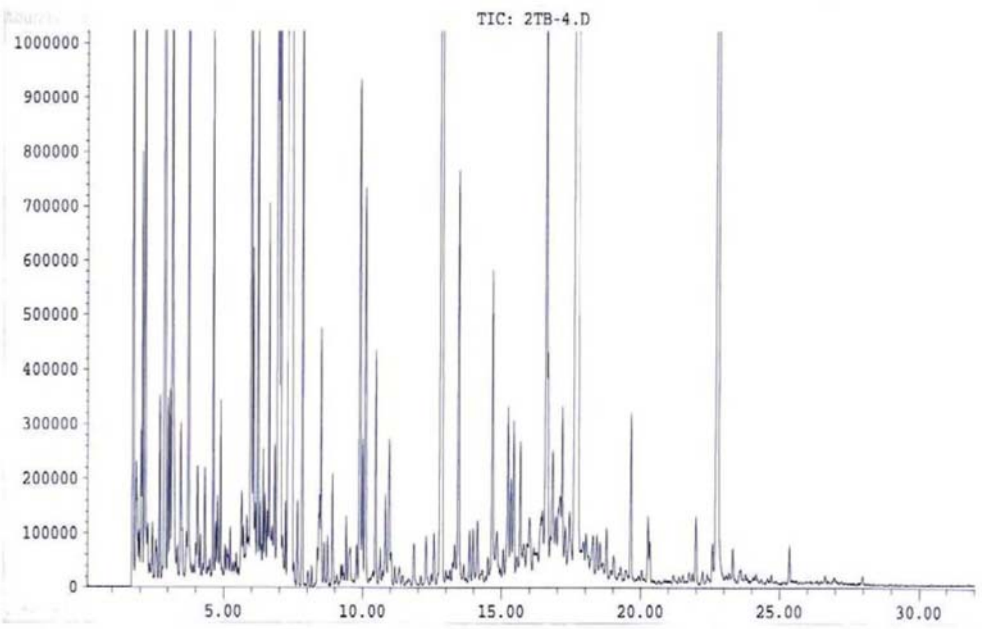

**FIGURE 2**. Total ion chromatogram of an air sample collected in a Tedlar bag, preconcentration volume 300 ml.

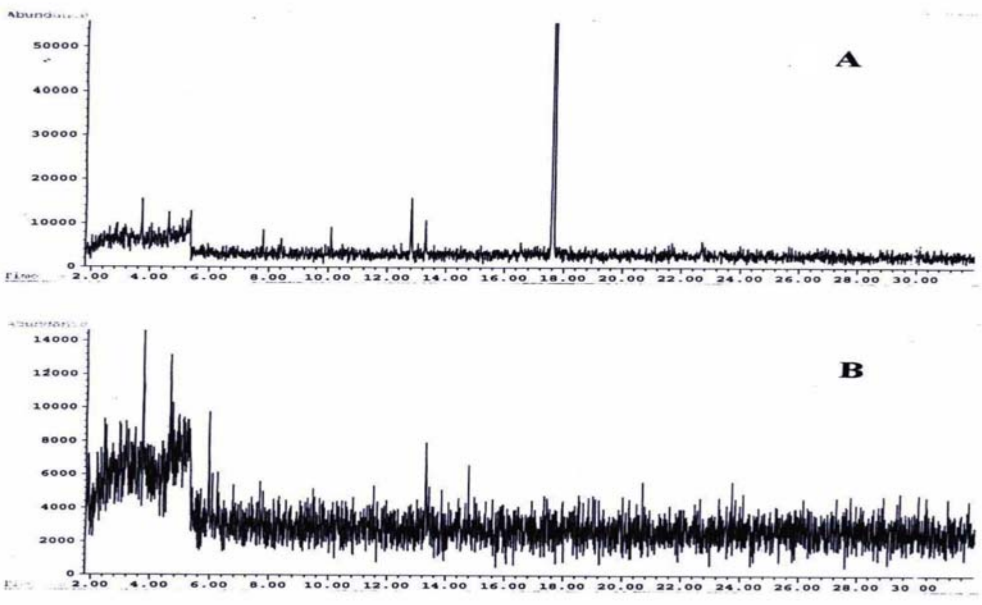

**FIGURE 3**. Comparison of total ion chromatograms of air samples collected outdoors (A) and indoors (B) in Tedlar bags, preconcentration volume 75 ml.

The GC-MS full-scan total ion chromatograms of air samples collected in an activated carbon tube and analyzed by a GC-MS without a cryogenic preconcentrator are shown in Fig. 4. It is not surprising to find that a very big peak, which has a relative longer retention time (about 26 min), appears in the chromatogram. Unfortunately, library search results from GC-MS cannot give positive identifications of the UVOC, but they do give a strong indication of the existence of the UVOC in Kuwait's ambient air.

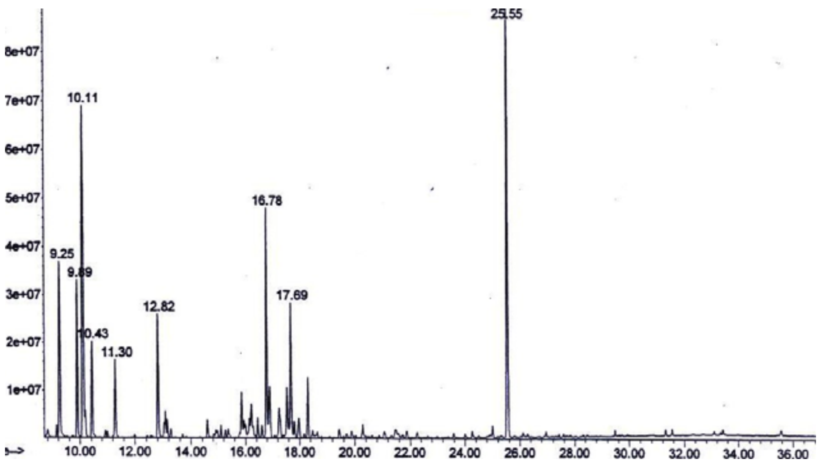

**FIGURE 4**. Total ion chromatogram of air sample collected by an activated carbon tube.

Tedlar bags utilize a lightweight, patented single fitting of inert polypropylene that combines the hose/valve and the septum holder into one compact fitting. A SUMMA canister is a stainless steel vessel that has had the internal surfaces specially passivated using a special process. This process combines an electropolishing step with chemical deactivation in order to produce a surface that is chemically inert. Actually, it is very difficult to deactivate all active points inside the canister's internal surface. The remaining active points will chemically or physically react with some compounds, which might be the reason to explain what we found in samples stored in canisters.

As described above, Kuwait belongs to a desert climate. Some photochemical reactions may produce special VOCs. It is necessary to further study this issue in Kuwait. This study also indicates that in order to obtain a more accurate picture of VOCs in a selected location, using different sampling technologies can avoid unnecessary mistakes.

## The Use of Internal Standard

It has been noticed that the analytical deviations among replicated results were large when using GC-MS equipped with a preconcentrator. In order to improve the analytical data quality, an add-in internal standard to Tedlar bags was studied. The procedure included simply using a syringe to add the internal standard to Tedlar bags containing air samples and VOC standards. The operation is easy and flexible. Our experiments found that adding an internal standard into air samples in Tedlar bags can greatly help to improve the data quality. As described above, using a syringe can easily introduce the internal standard into a Tedlar bag. However, there are some difficulties to adding an internal standard to a SUMMA canister by syringe at this moment. Table 2 lists the comparisons of relative deviations of analytical results for several VOCs. It can be seen that after correction of the peak areas based on a correction factor (CF) derived from the internal standard peak areas at different analyses, the quality of the duplicate results has been greatly improved. The average relative deviations change from 19% (no correction) to 4% (with correction). The CF is obtained as follows.

$$CF = PA1/PA2 \quad (1)$$

where CF – correction factor, PA1 – internal standard peak area in the first analysis, and PA2 – internal standard peak area in the second analysis. The corrected area (CPA2) for the second analysis is obtained by Equation 2.

$$CPA2 = CF \times PA2 \quad (2)$$

**TABLE 2**
**Comparison of Relative Divisions in Duplicate Analysis of Several VOCs With and Without Internal Standard**

| VOC ID | Peak Area in 1st Run | Peak Area in 2nd Run | Corrected Peak Area in 2nd Run | Relative Deviation Without Correction (%) | Relative Deviation After Correction (%) |
|---|---|---|---|---|---|
| Internal STD | 8648348 | 6171565 | (8648348) | | |
| 1 | 701020 | 401024 | 561955 | 43 | 20 |
| 2 | 235183 | 186654 | 261558 | 21 | 11 |
| 3 | 197965 | 132067 | 185065 | 33 | 7 |
| 4 | 209875 | 152944 | 214320 | 27 | 2 |
| 5 | 832409 | 607262 | 850956 | 27 | 2 |
| 6 | 197055 | 132067 | 185065 | 33 | 6 |
| 7 | 690236 | 530503 | 743394 | 23 | 8 |
| | | Average Relative Deviation: | | 30 | 8 |

The relative deviation (RDEV) is obtained using Equation 3.

$$RDEV = 100\frac{/(PA1 - PA2)/}{PA1} \quad (3)$$

Actually, using internal standard in calibration curve preparation can also improve the quality of the curve. For example, Table 3 lists three-level (5, 25, and 50 ppb) VOC standard peaks in three analyses. The peak areas of the internal standard in the three analyses were very different. Therefore, it is not surprising to find that each VOC's peak area had no reasonable relations compared to its standard values (see Fig. 5).

We chose 36.E+6 as the internal standard peak area for all three-level concentration analyses and the corrected peak areas are listed in Table 4. It can be observed that after correction, the relation between the peak area value and the concentration level looks much more reasonable (see Fig. 6). Table 4 and Fig. 6 demonstrate the importance of using internal standard for accurately analyzing VOCs in air.

## Effects of Preconcentrated Air Sample Volumes to Analytical Results

From an analytical point of view, preconcentrating larger air sample volumes can absolutely decrease VOC detection limits. Unfortunately, there are some risks from the instrument itself. Large preconcentration volumes might exceed the capacities of GC-MS or the preconcentrtor. For example, the Model 7000 preconcentrator declares that it can handle up to a 2000-ml air sample. Our study found that the proper volume must be obtained from tests. We used our three-level standards to study different preconcentration volumes at 150, 200, 300, and 450 ml. Then, each VOC peak's areas obtained at 25 and 50 ppb were divided by the peak area obtained at 5 ppb, respectively. The average peak area ratios for all VOCs at different standard levels are listed in Table 5. It can be found that 300 ml was the highest preconcentration volume for most of the VOCs in the three standard levels. But when using 450 ml as the preconcentration volume, the ratios already dropped to about 85% of the theoretical ratio. The above findings indicate that the VOC concentrations in an air sample are a key factor for selecting a proper preconcentration volume. For accurate results, conducting several analyses using different preconcentration volumes will help to improve analytical data quality. It is also noticed that some VOCs (such as 1,2,3-trimethylbenzene) showed almost the same peak areas at 5-, 25-, and 50-ppb levels in the studied preconcentration volumes, which indicates that even at 150 ml, the system was already saturated for 1,2,3-trimethylbenzene.

**TABLE 3**
**Comparison of VOCs and Internal Standard Peak Areas at Three Concentration Levels**

| Number | VOC ID | Original Peak Area | | |
|---|---|---|---|---|
| | | 5 ppb | 25 ppb | 50 ppb |
| | Internal standard | 36350888 | 8604547 | 16874926 |
| 1 | Methanechlorodifluoro | 3241465 | 1440118 | 11394498 |
| 2 | Butane | 1657076 | 922832 | 5759330 |
| 3 | Butane, 2-methyl | 2642256 | 2042028 | 12092306 |
| 4 | Pentane | 2183484 | 1861501 | 10502774 |
| 5 | 2,3-Pentadiene | 3455701 | 2039580 | 9410610 |
| 6 | Ethane, 1,1,2-trichlo | 8596797 | 7430762 | 48840642 |
| 7 | Methanedichloro | 2315715 | 2010836 | 10651796 |
| 8 | Pentane, 2-methyl | 2575466 | 2852925 | 14781786 |
| 9 | Cyclopentane | 1620627 | 1814409 | 8842649 |
| 10 | 1-Pentene, 2-methyl | 2392801 | 2987193 | 14938405 |
| 11 | Hexane | 2457312 | 3111803 | 15863266 |
| 12 | 2-Butanone | 1063975 | 1552708 | 5981736 |
| 13 | Chloroform | 5693086 | 9049861 | 46501868 |

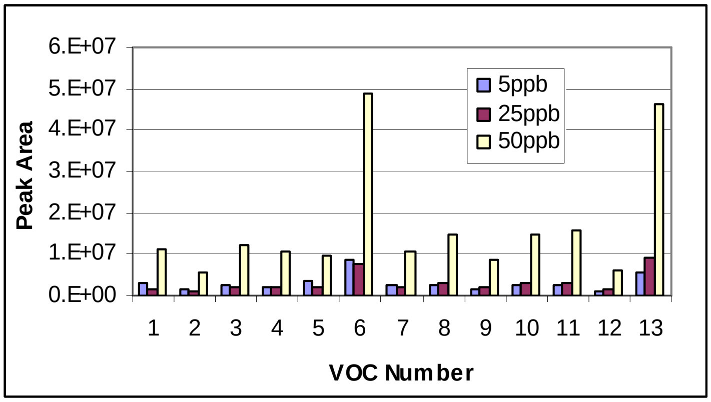

**FIGURE 5**. Uncorrected VOC peak areas comparison at three-level concentrations.

Our study also showed that there was a risk to use different preconcentration volumes for standards and air samples. Table 6 lists some VOC peak area comparisons at different preconcentration volumes. All the areas have been corrected based on the internal standard. It can be found that the peak areas are not proportional to the preconcentration volumes. For example, the area ratio between volume 150 to 50 ml should be 3, but the actual ratios are from 3 to 5. Thus, if using 50 ml as preconcentration volume for standard and using 150 ml as preconcentration volume for air samples, relative standard deviations for some VOCs such as VOCs with area rations 5 will be very large. Therefore, using same volumes for calibration standards and air samples can improve analytical data quality.

**TABLE 4**
**Comparison of Corrected VOCs Peak Areas and Internal Standard Peak Areas at Three Concentration Levels**

| Number | VOC ID | Corrected Peak Area | | |
|---|---|---|---|---|
| | | 5 ppb | 25 ppb | 50 ppb |
| | Internal standard | 36000000 | 36000000 | 36000000 |
| 1 | Chlorodifluoromethane | 3209050 | 6019693 | 24270281 |
| 2 | Butane | 1640505 | 3857438 | 12267373 |
| 3 | Butane, 2-methyl | 2615833 | 8535677 | 25756612 |
| 4 | Pentane | 2161649 | 7781074 | 22370909 |
| 5 | 2,3-Pentadiene | 3421144 | 8525444 | 20044599 |
| 6 | Ethane, 1,1,2-trichlo | 8510829 | 31060585 | 104030567 |
| 7 | Dichloromethane | 2292558 | 8405294 | 22688325 |
| 8 | Pentane, 2-methyl | 2549711 | 11925227 | 31485204 |
| 9 | Cyclopentane | 1604421 | 7584230 | 18834842 |
| 10 | 1-Pentene, 2-methyl | 2368873 | 12486467 | 31818803 |
| 11 | Hexane | 2432739 | 13007337 | 33788757 |
| 12 | 2-Butanone | 1053335 | 6490319 | 12741098 |
| 13 | Chloroform | 5636155 | 37828419 | 99048979 |

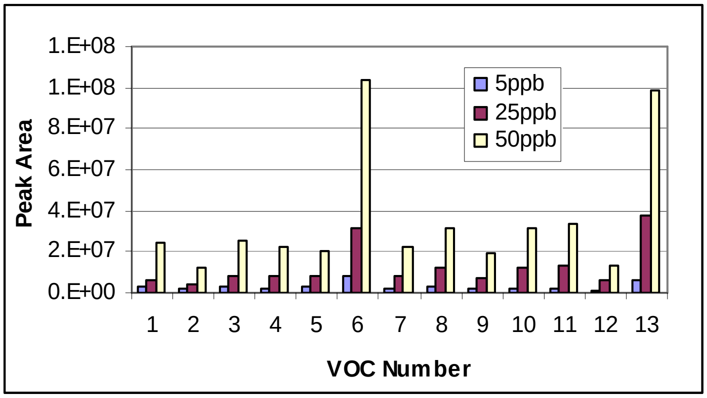

**FIGURE 6**. Corrected VOC peak areas comparison at three-level concentrations.

**TABLE 5**
**Comparisons of Average Peak Ratios at Different Preconcentration Volumes**

| Preconcentration Volume (ml) | Average Peak Area Ratio of 25 ppb/5 ppb | Average Peak Area Ratio of 50 ppb/5 ppb |
|---|---|---|
| 150 | 4.55 | 9.20 |
| 300 | 4.29 | 9.12 |
| 450 | 4.20 | 8.54 |

**TABLE 6**
**Comparison of Several VOC Peak Areas at Different Preconcentration Volumes**

| VOC Number | 50 ml (Cor. Area) | 150 ml (Cor. Area) | 300 ml (Cor. Area) | Area Ratio (150 ml/50 ml) | Area Ratio (300 ml/50 ml) |
|---|---|---|---|---|---|
| 1 | 264867 | 892845 | 1375196 | 3 | 5 |
| 2 | 115618 | 434844 | 742237 | 4 | 6 |
| 3 | 146620 | 739170 | 1362560 | 5 | 9 |
| 4 | 126760 | 619799 | 1109848 | 5 | 9 |
| 5 | 224276 | 652942 | 1152989 | 3 | 5 |
| 6 | 575311 | 2817176 | 5370401 | 5 | 9 |
| 7 | 181048 | 618710 | 1145457 | 3 | 6 |
| 8 | 201831 | 808736 | 1485582 | 4 | 7 |
| 9 | 112331 | 523807 | 992741 | 5 | 9 |
| 10 | 178888 | 803416 | 1594996 | 4 | 9 |
| 11 | 194841 | 779392 | 1564041 | 4 | 8 |
| 12 | 81697 | 332093 | 703246 | 4 | 9 |
| 13 | 444177 | 2199468 | 4855595 | 5 | 11 |
| 14 | 250570 | 1078752 | 2215903 | 4 | 9 |
| 15 | 240958 | 1145566 | 2672100 | 5 | 11 |
| 16 | 578807 | 2510966 | 5003997 | 4 | 9 |

## CONCLUSIONS

Kuwait experiences desert climatic weather. Due to the extreme hot and dry conditions in this country, our systematic study of analysis of VOCs in air by using GC-MS with a cryogenic trap has found useful information. Using Tedlar bags and SUMMA canisters to collect VOCs in the ambient air had different results; some VOCs had substantially decreased their concentrations in canisters. Different cryogenic preconcentration volumes in the trap could affect the analytical data quality, calibration curves for different compounds, and replicated results. The study concludes that to choose a sample container properly is a key element for successfully completing a project. Because GC-MS with a cryogenic trap is a dynamic system, replicated results normally showed poor agreement. The add-in internal standard to Tedlar bags containing standards and air samples can help to improve the replicated results.

## ACKNOWLEDGMENTS

We would like to express our appreciation and thanks to Mr. Niser Ahmed and his colleagues from the Central Analytical Laboratory of KISR for extracting the activated carbon tube and analyzing the extract by using their GC-MS system.
